# Exploration of the common genetic landscape of COVID-19 and male infertility

**DOI:** 10.3389/fimmu.2023.1123913

**Published:** 2023-03-20

**Authors:** Yinwei Chen, Taotao Sun, Kang Liu, Penghui Yuan, Chang Liu

**Affiliations:** ^1^ Reproductive Medicine Center, Tongji Hospital, Tongji Medical College, Huazhong University of Science and Technology, Wuhan, Hubei, China; ^2^ Department of Urology, Tongji Hospital, Tongji Medical College, Huazhong University of Science and Technology, Wuhan, Hubei, China; ^3^ Department of Urology, the First Affiliated Hospital of Zhengzhou University, Zhengzhou, Henan, China; ^4^ Reproductive Medicine Center, Nanjing Drum Tower Hospital, The Affiliated Hospital of Nanjing University Medical School, Nanjing, Jiangsu, China

**Keywords:** COVID-19, SARS-CoV-2, male infertility, DNA damage, immune

## Abstract

**Background:**

COVID-19 has spread widely across continents since 2019, causing serious damage to human health. Accumulative research uncovered that SARS-CoV-2 poses a great threat to male fertility, and male infertility (MI) is a common comorbidity for the COVID-19 pandemic. The aim of the study was to explore the cross-talk molecular mechanisms between COVID-19 and MI.

**Materials and methods:**

A total of four transcriptome data regarding COVID-19 and MI were downloaded from the Gene Expression Omnibus (GEO) repository, and were divided for two purposes (initial analysis and external validation). Differentially expressed genes (DEGs) analysis, GO and pathway annotation, protein-protein interaction (PPI) network, connectivity ranking, ROC analysis, immune infiltration, and translational and post-translational interaction were performed to gain hub COVID-19-related DEGs (CORGs). Moreover, we recorded medical information of COVID-19 patients with MI and matched healthy controls, and harvested their sperm samples in the university hospital. Expressions of hub CORGs were detected through the qRT-PCR technique.

**Results:**

We identified 460 overlapped CORGs in both the COVID-19 DEGs and MI DEGs. CORGs were significantly enriched in DNA damage and repair-associated, cell cycle-associated, ubiquitination-associated, and coronavirus-associated signaling. Module assessment of PPI network revealed that enriched GO functions were closely related to cell cycle and DNA metabolism processes. Pharmacologic agent prediction displayed protein-drug interactions of ascorbic acid, biotin, caffeine, and L-cysteine with CORGs. After connectivity ranking and external validation, three hub CORGs (ENTPD6, CIB1, and EIF3B) showed good diagnostic performance (area under the curve > 0.75). Subsequently, three types of immune cells (CD8+ T cells, monocytes, and macrophages M0) were dominantly enriched, and 24 transcription factor-CORGs interactions and 13 miRNA-CORGs interactions were constructed in the network. Finally, qRT-PCR analysis confirmed that there were significant differences in the expression of hub CORGs (CIB1 and EIF3B) between the patient and control groups.

**Conclusion:**

The present study identified and validated hub CORGs in COVID-19 and MI, and systematically explored molecular interactions and regulatory features in various biological processes. Our data provide new insights into the novel biomarkers and potential therapeutic targets of COVID-19-associated MI.

## 1 Introduction

Coronavirus disease 2019 (COVID-19) pandemic was first reported in December 2019, in Wuhan. A highly contagious coronavirus, severe acute respiratory syndrome coronavirus 2 (SARS-CoV-2), caused this disease. Up to 2022 December, it resulted in more than 600 million cumulative cases and more than 6 million cumulative deaths globally (https://covid19.who.int/), posing a persistent threat to human health. The main manifestation of COVID-19 included fever, cough, shortness of breath, loss of taste or smell, and so on (1). In addition to the above common symptoms, the impact of COVID-19 on human fertility is also receiving increasing attention. Specifically, the association between COVID-19 and diminished male fertility has been revealed in various countries ^2^. However, the study on male fertility and COVID-19 was still limited.

Male infertility (MI) is becoming a serious medical issue on a global scale. Approximately 10-15% of couples suffered from infertility worldwide, and male causes are responsible for 50% of cases (2, 3). In the recent three years, cumulative evidence uncovered the tight relationship between the COVID-19 pandemic and male infertility (4). SARS-CoV-2 has been confirmed to exist in semen specimens and seminiferous tubes in the testes of male COVID-19 patients (5). Koç et al. (6) and Mannur et al. (7) both suggested that COVID-19 had an unfavorable influence on the semen parameters in infertile men, including sperm count, motility, and morphology. Moreover, it is reported that COVID-19 patients in the recovery state had impaired sperm motility and higher sperm DNA fraction index (8).

Angiotensin-Converting Enzyme 2 (ACE2), the cellular receptor of SARS-CoV-2, serves as the main component of the mechanism for access to human host cells (9). Spike protein of SARS-CoV-2 binds to ACE2 in the cell membrane. Then, transmembrane serine protease type II (TMPRSS2) cleaves spike protein, which causes the cell membrane to fuse and allows for viral penetration. It is demonstrated that ACE2 and TMPRSS2 are extensively expressed in male urogenital organs, including testes, epididymis, and prostate gland (10, 11), which indicates that spermatogenesis is vulnerable to SARS-CoV-2 infection. Currently, the mechanisms underlying COVID-19-associated male infertility have attracted more attention. Two cohort studies found an evident increase in serum luteinizing hormone (LH) level, and a notable decrease in serum testosterone (T) level in patients with COVID-19 (8, 12), confirming the finding that SARS-CoV-2 replication contributes to damage in Leydig cells and further causes impaired T production (13). Xiong et al. showed that there were large numbers of CD3+ and CD68+ immunocytes in the interstitial cells and elevated apoptotic cells within seminiferous tubes in the testes of COVID-19 patients, suggesting the disturbances in immune regulation in the spermatogenic environment.

However, the specific mechanism of impaired spermatogenesis after SARS-CoV-2 infection remains largely unknown. In the era of genetic data, the rapid progression of microarray and high-throughput sequencing brings great convenience to interpreting the molecular mechanisms in the development of diseases. In the present study, we used multi-omics analysis to identify and validate hub COVID-19-related differentially expressed genes (CORGs) in both diseases of COVID-19 and MI. Multidimensional crosslinks of CORGs in signaling, drug target, immune response, and transcriptional and post-transcriptional regulation were systematically analyzed. Experimental validation was also performed on human specimens. The study would enhance our understanding of the pathogenesis of COVID-19-associated MI from the perspective of genetics.

## 2 Methods and materials

### 2.1 Data acquisition

Expression array data and high throughput sequencing data regarding “COVID-19”, “SARS-CoV-2”, and “male infertility” were screened and downloaded from the GEO database (https://www.ncbi.nlm.nih.gov/geo/). The GSE164805 dataset contained transcriptome data of peripheral blood mononuclear cells taken from COVID-19 patients as well as healthy controls. The GSE160749 dataset contained transcriptome data of sperm samples taken from patients with male infertility and healthy controls. To further improve our data’s validity and authenticity, we used the GSE147507 dataset (COVID-19) and GSE26881 dataset (male infertility, including oligospermia, asthenozoospermia, and teratozoospermia) as independent validation sets.

### 2.2 Identification of DEGs

To acquire the normalized expression matrixes, we used the R package “limma” (14) to convert the probe names, normalize the expression of each sample, and apply log2 transformation (if the original data was not transformed). For the RNA sequencing data (Count), we used the R packages “edgeR” (15) and “limma”. For all datasets, we set |log2 fold-change| (|log2FC|) > 0.5 and P value < 0.05 as the screening criteria to obtain CORGs. The display of DEGs was used by the R packages “ggplot2” (16) and “heatmap” (https://stat.ethz.ch/R-manual/R-devel/library/stats/html/heatmap.html). When we need to take the intersection of different gene sets, the R package “Venn diagram” (17) was utilized.

### 2.3 GO and pathway enrichment annotation

DAVID online tool (https://david.ncifcrf.gov/) was explored to provide functional classification, biochemical pathway maps, and conserved protein domain architectures implicated in the various physiological and pathological processes. Herein, we analyzed the significant signaling after uploading the CORGs. Three types of Gene Ontology (GO) terms, including biological process (BP), cellular component (CC), and molecular function (MF) were obtained (18). The requirements of screening the significant GO terms included gene number>2 and Ease < 1. For pathway analysis, we utilized the KEGG, REACTOME, and WikiPathway databases to acquire the most enriched pathways.

### 2.4 PPI network construction and module analysis

To comprehensively understand the interrelationship of CORGs, the protein-protein interaction (PPI) network construction was carried out using the Search Tool for the Retrieval of Interacting Genes (STRING) database (http://string-db.org). We removed CORGs with low connectivity according to the criteria of medium confidence > 0.4. The visualization of the PPI network was achieved with the help of Cytoscape software (version 3.7.1) (19). Module analysis was performed to obtain the gene clusters with more important values in the gene set of CORGs. We first calculated the module score by using the Molecular Complex Detection (MCODE) plugin in the Cytoscape software. Then, we selected the top three modules with the highest scores and downloaded the module composition of each module. GO and pathway analysis were used to obtain enriched biological processes and signaling alterations in each module using the Metascape online tool (http://metascape.org). Our cutoff in the Metascape was a P value < 0.01, a minimum count of 3, and an enrichment factor > 1.5.

### 2.5 Pharmacologic agent prediction

To explore pharmacologic agent candidates that predominantly target and influence CORGs, we used the DrugBank database (Version 5.0) to predict the pharmacologic agent candidates. The interaction network of CORGs and candidates were constructed in the NetworkAnalyst online tool (https://www.networkanalyst.ca/) according to the connectivity of each pharmacologic agent candidate. The visualization of the network was implemented *via* the Cytoscape software.

### 2.6 Detection of significant CORGs

With the use of the cytoHubba plugin in the Cytoscape software, different connectivity ranking calculation was applied to CORGs. The algorithms included MCC, EPC, BottleNeck, EcCentricity, Closeness, and Radiality. Only the significant CORGs with high scores were used for further investigation.

### 2.7 ROC analysis

Receiver operating characteristic(ROC)analysis is a well-established statistical method that illustrates the diagnostic ability of variables and assesses the accuracy of model predictions. Herein, we used ROC analysis to determine the diagnostic ability of significant CORGs in the COVID-19 dataset, MI dataset, and validation sets. The R package “timeROC” (https://cran.r-project.org/web/packages/timeROC/) (20) was used to perform the ROC analysis. We first selected the CORGs with the area under the curve (AUC) > 0.9 in the COVID-19 and MI datasets. Subsequently, we performed ROC analysis once again in the two independent validation sets (GSE147507 and GSE26881), only the CORGs with AUC > 0.75 in the validation sets were regarded as hub CORGs.

### 2.8 immune infiltration cell assessment

To mine the immunological influence in the progression of COVID-19 and MI, we explored the CIBERSORT tool (21) to analyze the composition level of different immune cells. With the use of the R packages “e1071”, “parallel”, and “preprocessCore”, we obtained the ratio of immune cells in each sample in both the COVID-19 and MI datasets. The differences between the two groups (COVID-19 vs. control, and MI vs. control) were evaluated by Wilcoxon’s test. The relevance of immune cells and hub CORGs was analyzed using Spearman’s test. The threshold for statistical significance was set at P < 0.05.

### 2.9 Transcriptional and post-transcriptional networks analysis

We performed the network analysis of hub CORGs and transcription factor (TF) (the transcriptional level), and hub CORGs and miRNA (the post-transcriptional level). The TF-CORGs interactions and miRNA-CORGs interactions were obtained from the RegNetwork repository (22). The visualization of the above interactions was achieved by the Cytoscape software.

### 2.10 Hub gene interaction

The interacting genes of hub CORGs were discovered using the web program GeneMANIA (http://genemania.org/) to create the hub CORGs-gene interaction network. These interacting genes should meet one or more of the following relationships: physical interaction, co-expression, predicted, co-localization, genetic interactions, pathway, and shared protein domains.

### 2.11 Disease association analysis

To explore which diseases hub CORGs are predominantly associated with, we analyzed the most enriched diseases for each gene in the Comparative Toxicogenomics Database (CTD, http://ctdbase.org/) (23). We focused on male reproductive system diseases, and the threshold for screening was set at an inference score > 40.

### 2.12 Participant recruitment

In our previous findings (24), we recruited a cohort of male COVID-19 patients and healthy controls attending Tongji hospital. After reviewing their medical records, we found seven healthy controls and seven patients with male infertility which manifested no pregnancy after unprotected sexual intercourse for more than one year, and different degrees of abnormal semen parameters (oligospermia, asthenozoospermia, and teratozoospermia) based on the WHO laboratory manual (25). For the diagnosis of COVID-19, real-time reverse transcriptase-polymerase chain reaction (RT-PCR) analysis of pharyngeal swab specimens was used to demonstrate that the patients were SARS-CoV-2 positive. Then, all patients were regarded as in the recovery state according to relieving symptoms and two consecutively negative testing for SARS-CoV-2. The New Coronavirus Pneumonia Prevention and Control Program (7th edition) issued by the National Health Commission of China, served as the basis for the diagnosis of COVID-19. The study received permission from Huazhong University of Science and Technology’s institutional research ethics committee. Each participant was provided with written informed consent, and all procedures followed the Helsinki Declaration.

### 2.13 Sperm processing

The clinical and demographic information was recorded at the participant’s hospital visit. For patients, their semen examinations were performed at the first visit during the recovered period, and the recovered time for the seven patients was all ≤ 100 days. The serum Follicle−stimulating hormone (FSH), luteinizing hormone (LH), total T, and estradiol (E_2_) concentrations were measured using a chemiluminescence immunoassay (Beckman Coulter, Fullerton, USA). Following the three to seven days of advised abstinence, freshly obtained semen was processed for 30 to 60 minutes at room temperature to liquefy it. Sperm examination was performed in strict accordance with the WHO laboratory manual (25). Purified sperm samples were obtained by centrifuging them for 20 minutes at 1000 g while using a Percoll gradient (Solarbio, Beijing, China). After washing them with PBS twice, the purified sperm samples were used for subsequent experiments.

### 2.14 qRT-PCR measurement

Following the manufacturer’s instructions, we extracted RNA from purified sperm samples using RNA-easyTM Isolation Reagent (Vazyme, Nanjing, China) and synthesize cDNA using the Hifair^®^ II 1st Strand cDNA Synthesis Kit (Yeasen, Shanghai, China). Herein, we diluted the cDNA to the proper concentrations, and performed quantitative real-time polymerase chain reaction (qRT-PCR) as previously described (26). Expression levels of hub CORGs were calculated after normalization of the reference gene *GAPDH*. The detailed information on primers used in the study is shown in **Supplementary Table 1**.

### 2.15 Immunohistochemical analysis

We retrieved the immunohistochemistry data from the Human Protein Atlas database (HPA) (https://www.proteinatlas.org/), and accessed the positive region of hub CORGs to identify the expression signature in testes.

### 2.16 Statistical analysis

The clinical and demographic data of participants and mRNA levels of hub CORGs were presented as mean ± standard deviation (SD). The comparisons were performed using Student’s t test in built-in algorithms of Graphpad software (LLC, San Diego, California USA). The threshold for statistical significance was set at P < 0.05.

## 3 Results

### 3.1 Discovery of overlapped CORGs

**Figure 1:**
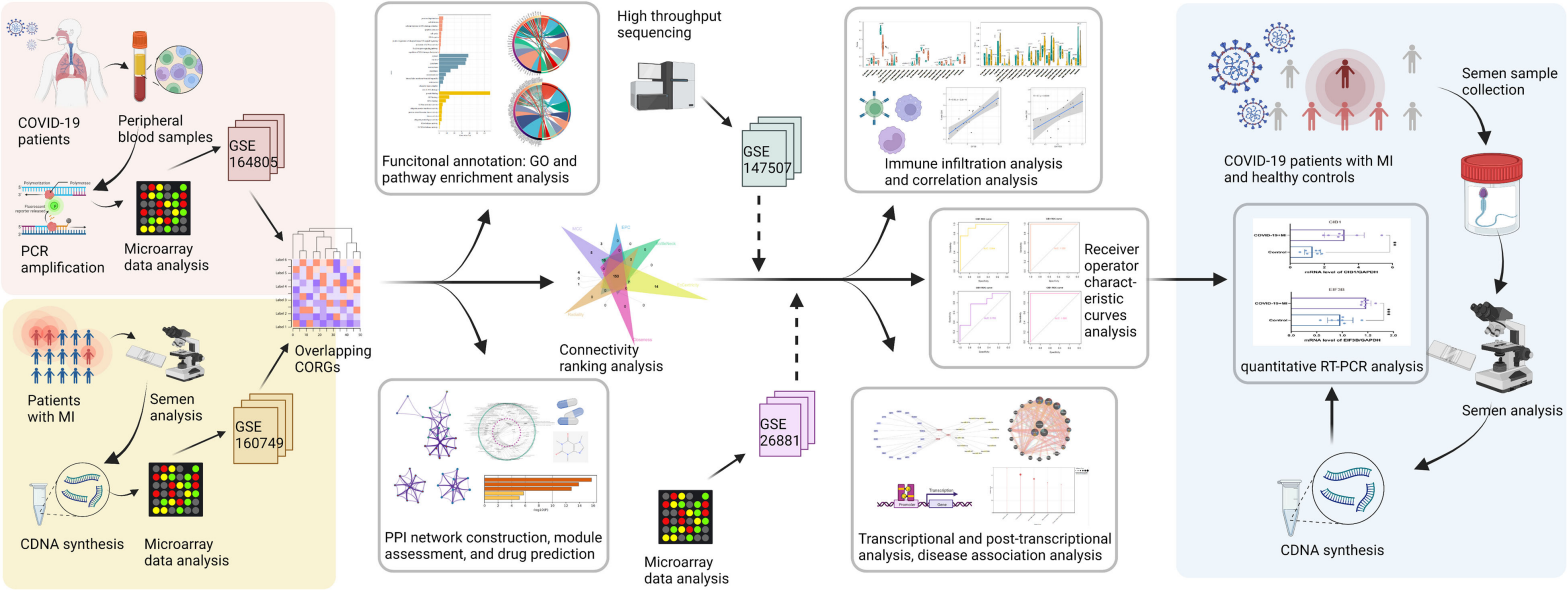
The methodical flowchart of the study. MI, male infertility; GSE, GEO Series; CORGs, COVID-19-related differentially expressed genes; GO, Gene Ontology; PPI, protein-protein interaction; ROC, receiver operating characteristic; PCR, polymerase chain reaction; quantitative RT-PCR, quantitative real-time polymerase chain reaction.

**Figure 2:**
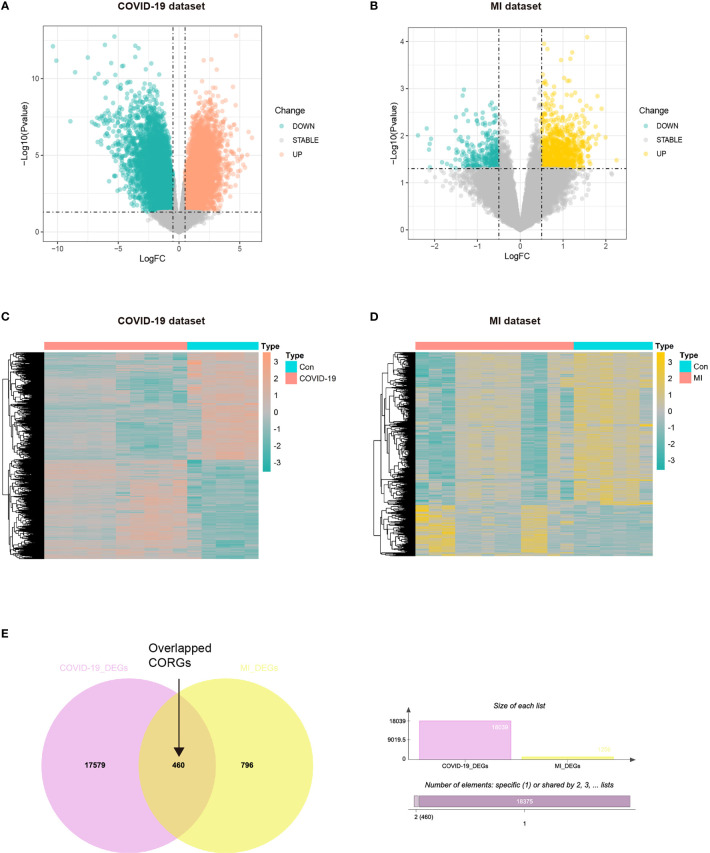
Acquisition of overlapped CORGs in the COVID-19 dataset (GSE164805) and MI dataset (GSE160749). **(A)** DEGs in the COVID-19 and control groups were shown in the Volcano plot, green represents downregulation, grey represents stability, and orange represents upregulation. **(B)** DEGs in the MI and control groups were shown, green represents downregulation, grey represents stability, and yellow represents upregulation. **(C)** DEGs were shown in the COVID-19 heatmap. **(D)** DEGs in the MI heatmap. **(E)** Venn diagram depicts overlapped CORGs in both the COVID-19 DEGs and MI DEGs. MI, male infertility; GSE, GEO Series; CORGs, COVID-19-related differentially expressed genes; DEGs, differentially expressed genes.

The study strategy was depicted in **Figure 1**. With the threshold of log2FC > 0.5, we obtained the DEGs taken from the COVID-19 dataset and MI dataset. The results showed that there were 944 upregulated DEGs and 312 downregulated DEGs between the COVID-19 and healthy control samples (**Figures 2A, C**), and there were 9372 upregulated DEGs and 8667 downregulated DEGs between the MI and control samples (**Figures 2B, D**). To obtain the common genes implicated in both the disease of COVID-19 and MI, we took the intersection of COVID-19 DEGs and MI DEGs, and a total of 460 overlapped CORGs were discovered in both DEGs sets (**Figure 2E**).

### 3.2 Functional annotation of CORGs

**Figure 3:**
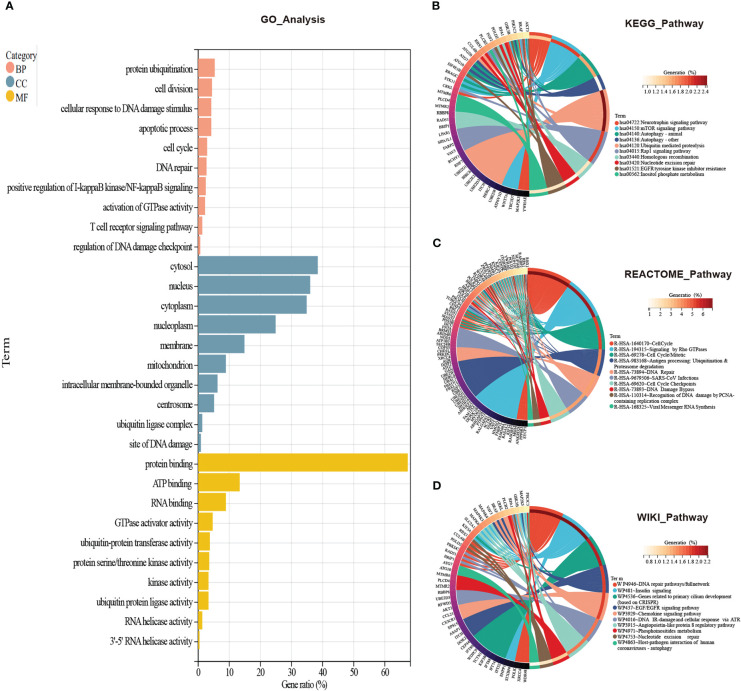
GO and pathway enrichment analysis of CORGs. **(A)** GO enrichment analysis of CORGs. Pathway enrichment analysis of CORGs in the KEGG database **(B)**, REACTOME database **(C)**, and WikiPathways database **(D)**. CORGs, COVID-19-related differentially expressed genes; GO, Gene Ontology; BP, biological process; CC, cellular component; MF, molecular function.

To figure out which signaling the CORGs predominantly participated in, we used four databases to perform GO analysis (GO database) and pathway analysis (KEGG, REACTOME, and WikiPathways databases). As shown in **Figure 3A**, for biological process, GO terms were mainly enriched in protein ubiquitination, cell division, cellular response to DNA damage stimulus, apoptotic process, cell cycle, and DNA repair. GO terms for cellular component were tightly related to cytosol, nucleus, cytoplasm, and nucleoplasm. GO terms for molecular function were focused on protein binding, ATP binding, RNA binding, GTPase activator activity, and ubiquitin-protein transferase activity.

In another three pathway databases, the results showed the overlapped CORGs were notably enriched in DNA damage and repair-associated (DNA repair pathways/full network, DNA IR-damage and cellular response *via* ATR, DNA Damage Bypass, Recognition of DNA damage by PCNA-containing replication complex, and DNA Repair), cell cycle-associated (Cell Cycle, Cell Cycle Mitotic, Cell Cycle Checkpoints), ubiquitination-associated (Ubiquitin mediated proteolysis, Antigen processing: Ubiquitination & Proteasome degradation), and coronavirus-associated (SARS-CoV Infections, Viral Messenger RNA Synthesis, and Host-pathogen interaction of human coronaviruses-autophagy) pathways (**Figures 3B–D**). Combining the above results, DNA damage and repair-associated, cell cycle-associated, and ubiquitination-associated signaling were most enriched, and these signaling may play an important role in the initiation and development of COVID-19 and MI.

### 3.3 Module assessment of PPI network

**Figure 4:**
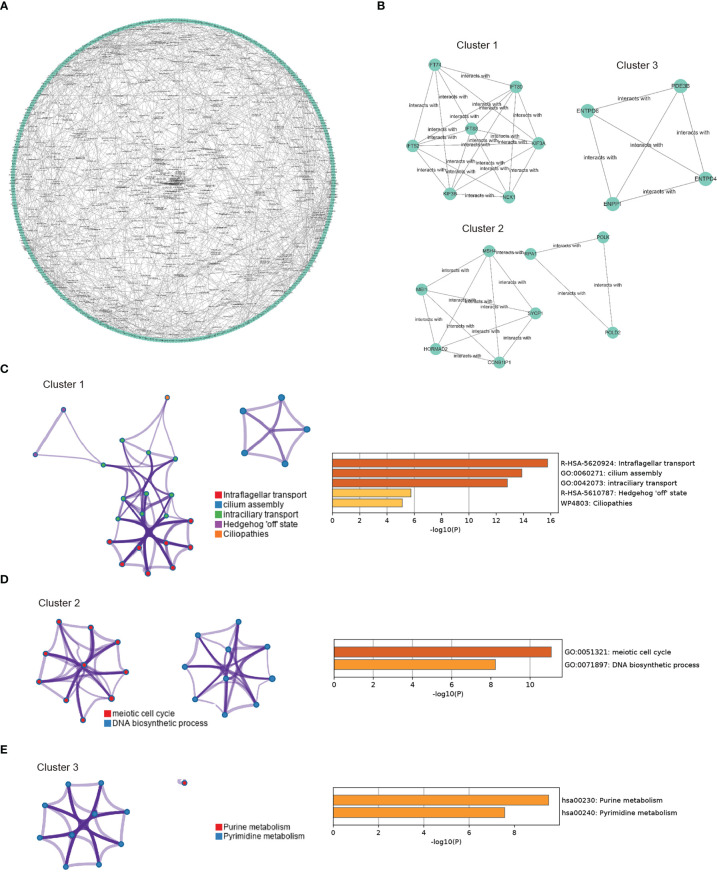
PPI network construction and module enrichment evaluation. **(A)** Protein-protein interaction network of CORGs using the STRING database. **(B)** Top three modules (Cluster 1-3) screened from PPI network using MCODE algorithm. **(C)** Go enriched-term interaction of the Cluster 1. **(D)** Go enriched-term interaction of the Cluster 2. **(E)** Go enriched-term interaction of the Cluster 3. Dots of the same colors indicate the same GO enriched-terms, and dots of size indicate the number of consensus genes in this GO term, the larger the number, the larger the size. PPI: protein-protein interaction; CORGs: COVID-19-related differentially expressed genes; GO: Gene Ontology.

In the next step, we aimed to investigate the interaction features of CORGs by using the STRING database. A total of 738 interactions were identified as shown in **Figure 4A**. Among all the interactions, we found that three genetic modules had the top highest density score, indicating the stronger level of interaction of CORGs in the three modules (**Figure 4B**). The enriched GO functions for Module one were involved in intraflagellar transport, cilium assembly, and intraciliary transport. GO functions for Module two had a close relationship with meiotic cell cycle and DNA biosynthetic processes. GO functions for Module three captured purine metabolism and pyrimidine metabolism (**Figures 4C–E**). The functional annotation confirmed the role of cell cycle and DNA metabolism once again.

### 3.4 Pharmacologic agent prediction

**Figure 5:**
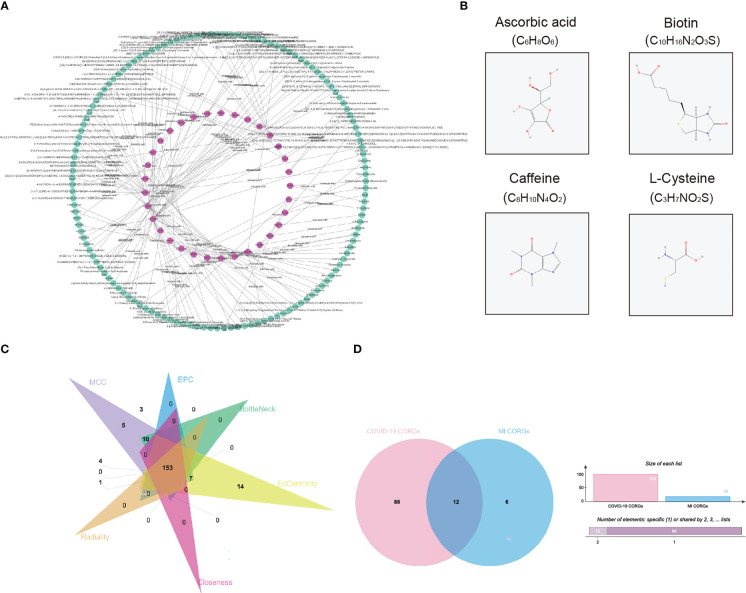
Pharmacologic agent prediction and connectivity ranking analysis. **(A)** Pharmacologic agent candidate-CORGs interaction network. **(B)** Two dimensional structures of pharmacologic agent candidates (ascorbic acid, biotin, caffeine, and L-cysteine) with supporting evidence in Pubmed. **(C)** Venn diagram depicts the overlapped CORGs in the connectivity ranking analysis (MCC, EPC, BottleNeck, EcCentricity, Closeness, and Radiality). **(D)** Venn diagram depicts the significant overlapped CORGs with AUC > 0.9 in both the COVID-19 dataset and MI dataset. CORGs, COVID-19-related differentially expressed genes; MI, male infertility.

To explore the pharmacologic agent candidates which may target the overlapped CORGs, we utilized prediction algorithm in the DrugBank database, and we harvested 135 pharmacologic agent candidates and 137 interactions with CORGs (**Figure 5A**). Among these candidates, we mined their therapeutic effects in Pubmed. Only four candidates (ascorbic acid, biotin, caffeine, and L-cysteine) had favorable effects on male infertility with *in vitro* or *in vivo* evidence, and their two-dimensional structures were shown in **Figure 5B**. It was implied that the four pharmacologic drugs have a good medical value in COVID-19-associated MI.

### 3.5 Screening for significant CORGs

Considering the large number of CORGs, we used connectivity ranking analysis and ROC analysis to narrow down the gene number. After implementing six connectivity ranking algorithms, we found 153 CORGs with high scores in **Figure 5C**. Moreover, ROC analysis showed that there were 100 CORGs with AUC > 0.9 in the COVID-19 datasets, and there were 18 CORGs with AUC > 0.9 in the MI datasets (**Supplementary Table 2**). A total of twelve significant CORGs (RNF7, UBE2G1, ATG7, AKT3, STIL, PDE3B, ENTPD6, EIF3B, ZEB1, RBP4, C2orf69, CIB1) showed AUC > 0.9 in both datasets, suggesting that these genes have higher research potentials (**Figure 5D**).

### 3.6 External validation of hub CORGs

**Figure 6:**
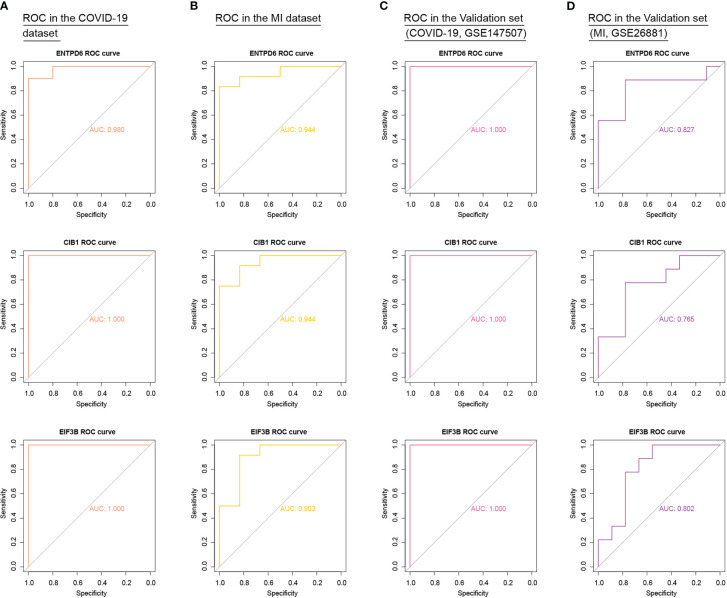
External validation of diagnostic values of three hub CORGs (ENTPD6, CIB1, and EIF3B) in COVID-19 dataset, MI dataset, and two independent validation sets. **(A)** ROC results of three hub CORGs in the COVID-19 dataset (AUC > 0.75). **(B)** ROC results of three hub CORGs in the MI dataset (AUC > 0.75). **(C)** ROC results of three hub CORGs in a validation set (COVID-19, GSE147507, AUC > 0.75). **(D)** ROC results of three hub CORGs in a validation set (MI, GSE26881, AUC > 0.75). CORGs, COVID-19-related differentially expressed genes; MI, male infertility; ROC, receiver operating characteristic; AUC, area under the curve.

To add authority and persuasion of significant CORGs, we examined the diagnostic value of twelve CORGs in other independent transcriptome data. For further validation, we first obtained 3129 DEGs between the COVID-19 and control samples in the COVID-19 dataset (GSE147507). Similarly, 441 DEGs were identified between the MI and control samples in the MI dataset (GSE26881) (**Supplementary Figure 1**). Next, we performed ROC analysis to validate the diagnostic effectiveness of CORGs in the above validation sets. As shown in **Figure 6**, it was revealed that three hub CORGs (ENTPD6, CIB1, and EIF3B) had good diagnostic performance to discriminate COVID-19/MI and control samples (AUC > 0.75). These hub CORGs were well verified in external transcriptome data and may have the potential as biomarkers for COVID-19-associated MI.

### 3.7 Immune infiltration analysis

**Figure 7:**
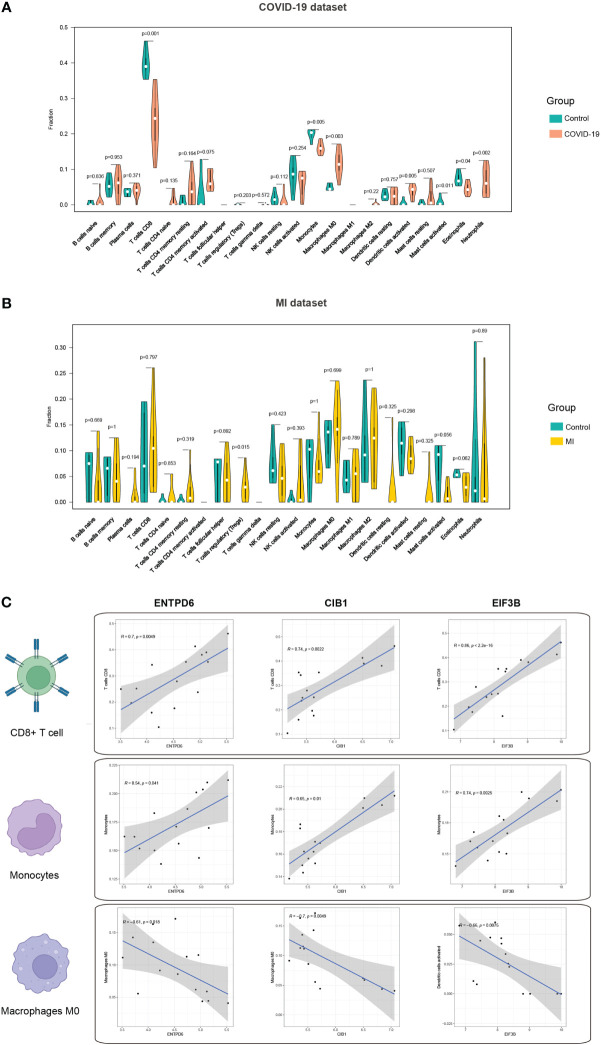
Immune infiltration profile of three hub CORGs. **(A)** Violin plot depicts significant multiple immune cells between the COVID-19 and control groups. **(B)** Violin plot depicts significant multiple immune cells between the MI and control groups. **(C)** Correlation analysis of hub CORGs (ENTPD6, CIB1, and EIF3B) and significant immune cells (CD8+ T cell, monocyte, and macrophages M0). CORGs, COVID-19-related differentially expressed genes; MI, male infertility.

Activation of immune response is inevitable in patients with COVID-19, thus, we mined the immune infiltration landscape in COVID-19 and MI based on genomic data. We first focused on the difference in immune composition between the patient and control groups. It is found that there were significant differences in the levels of CD8+ T cells, monocytes, macrophages M0, dendritic cells activated, mast cells activated, eosinophils, and neutrophils (**Figure 7A**). Furthermore, the level of regulatory T cells (Tregs) in the MI group was remarkedly different from the control group (**Figure 7B**). In the subsequent step, we analyzed the correlation degree between the immune cells and hub CORGs. Correlation results indicated that hub CORGs (ENTPD6 and CIB1) were positively related to the level of significant immune cells, while hub CORG (EIF3B) was negatively related. The detailed interrelationship was as follow: the level of CD8+ T cells (ENTPD6, r = 0.7, p = 0.0049; CIB1, r = 0.74, p = 0.0022; EIF3B, r = 0.86, p < 2.2 e^-16^), the level of monocytes (ENTPD6, r = 0.54, p = 0.041; CIB1, r = 0.65, p = 0.01; EIF3B, r = 0.74, p = 0.0025), and the level of macrophages M0 (ENTPD6, r = -0.61, p = 0.018; CIB1, r = -0.7, p = 0.0049; EIF3B, r = -0.66, p = 0.0075) (**Figure 7C**). These data suggested that hub CORGs may be implicated in affecting the sperm parameters in COVID-19 patients *via* disturbance of CD8+ T cells, monocytes, and macrophages M0.

### 3.8 Transcriptional and post-transcriptional signature

**Figure 8:**
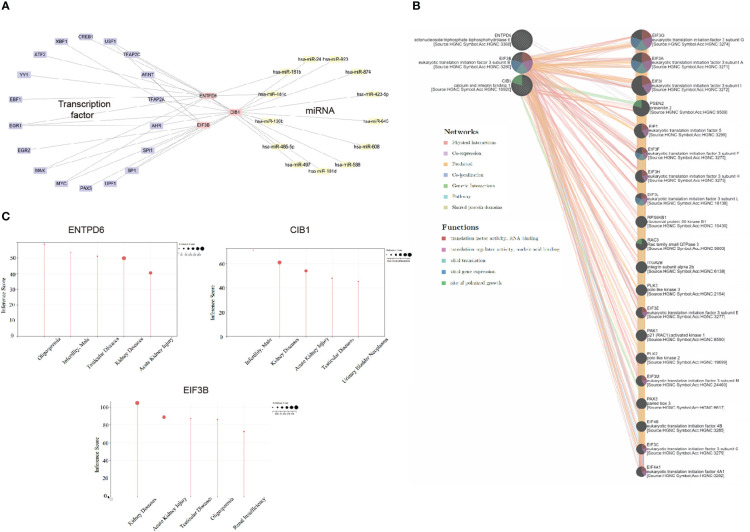
Transcriptional and post-transcriptional interaction, gene-gene interaction, and disease association analysis. **(A)** Transcriptional and post-transcriptional interaction network (including transcriptional factor and miRNA) visualized by Cytoscape. **(B)** Gene-gene interaction analysis using GeneMANIA. **(C)** Disease association analysis using CTD. CORGs, COVID-19-related differentially expressed genes; CTD, Comparative Toxicogenomics Database.

In this part, we investigated the upregulation and downregulation of hub CORGs. Therefore, we characterized the profile of interacting TFs and miRNAs. In **Figure 8A**, 24 TF-CORGs interactions and 13 miRNA-CORGs interactions were shown. Among all the interacting TFs, ARNT, EGR1, MAX, MYC, and USF1 were predicted to target at least two hub CORGs. For interacting miRNAs, 13 miRNAs were shown to have the potential to regulate the post-transcriptional level of hub CORGs, including hsa-miR-24, hsa-miR-181b, hsa-miR-181c, hsa-miR-130b, hsa-miR-485-5p, hsa-miR-497, hsa-miR-181d, hsa-miR-588, hsa-miR-608, hsa-miR-645, hsa-miR-423-5p, hsa-miR-874, and hsa-miR-923, which deserved further exploration in COVID-19 and MI.

### 3.9 Hub CORGs-gene interaction and disease association

With the use of the GeneMANIA program, we constructed the hub CORGs-gene interaction network (**Figure 8B**). Moreover, we found that this network was tightly relevant to the viral translation, viral gene expression, and site of polarized growth (false discovery rate < 0.05). It suggested that hub CORGs may mediate cellular viral replication and damage. For disease association, three hub CORGs strongly impacted diseases of the male reproductive system. Oligospermia, testicular diseases, and male infertility were highlighted in the diseases of the male reproductive system with an inference score > 40 (**Figure 8C**), which suggested the close association of hub CORGs in MI-associated illnesses.

### 3.10 Clinical characteristics of COVID-19 patients with MI

**Figure 9:**
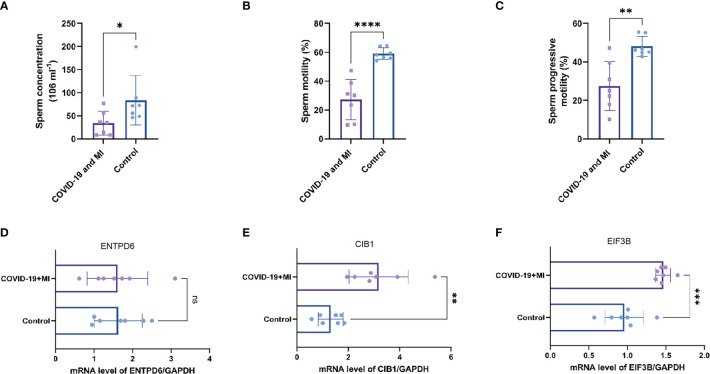
Patient’s semen comparisons and expression analysis of three hub CORGs. The levels of sperm concentration **(A)**, sperm motility **(B)**, and progressive motility **(C)** between the patient (COVID-19 and MI) and control groups. The mRNA levels of ENTPD6/GAPDH **(D)**, CIB1/GAPDH **(E)**, and EIF3B/GAPDH **(F)** between the patient and control groups. * indicates P < 0.05, ** indicates P < 0.01, *** indicates P < 0.001, *** indicates P < 0.0001. CORGs, COVID-19-related differentially expressed genes; MI, male infertility.

As shown in **Supplementary Table 3**, seven patients with male infertility had varying degrees of oligospermia, asthenozoospermia, and teratozoospermia. During their recovery period, they had normal serum hormone levels and no medical history of varicocele, genitourinary tract inflammation and infection, scrotal trauma, and so on. In comparison to the control group, the sperm concentration, motility, and progressive motility were significantly lower in the patient group (P < 0.05, **Figures 9A–C**).

### 3.11 Experimental detection of hub CORGs

To enhance authority and stringency, qRT-PCR was utilized to measure the mRNA levels of hub CORGs between the COVID-19 patients with MI and healthy controls. In **Figures 9D–F**, the results of qRT-PCR suggested that notable changes were observed in the mRNA levels of CIB1 and EIF3B in the patient group than those in the control group (P < 0.01 and P < 0.001, respectively). However, no pronounced alteration was observed in the mRNA of ENTPD6 between the two groups. Furthermore, immunohistochemical data showed that it is hard to detect the positive area of CIB1 in seminiferous tubules and interstitial region of testes. As for EIF3B, moderate positive blots were seen in spermatogenic and Sertoli cell in seminiferous tubules and Leydig cell in interstitial region (**Supplementary Figure 2**). It is noted that two hub genes (CIB1 and EIF3B) had the significant change pattern in the sequencing findings and qRT-PCR results, emphasizing the critical genetic links of CIB1 and EIF3B between COVID-19 and MI.

## 4 Discussion

In today’s world, COVID-19 pandemic and male infertility have been significant issues with regard to human health. Their close interrelationship further contributes to the deterioration of male fertility. Current investigation was predominantly focused on the cohort study regarding the alterations in semen parameters, there are few studies related to pathogenic mechanisms. Thus, this study was grounded in the genetic profile and signal signature in the two diseases. We systematically identified and validated hub CORGs, and explored crosslinks of CORGs and biological function in a multidimensional manner. Clinical characteristics and sperm profile of COVID-19 patients with MI was also recorded for validation of hub CORGs.

In this study, a total of 460 overlapped CORGs were identified based on gene sets of 18039 COVID-19 DEGs and 1256 MI DEGs. The number of overlapped CORGs accounts for approximately one-third of MI DEGs. GO and pathway analysis uncovered that these CORGs were conspicuously implicated in DNA damage and repair-associated, cell cycle-associated, and ubiquitination-associated signaling. Sperm DNA fragmentation is often present in abnormal semen results, and has a good potential for prediction of aberrant fertilization in assisted reproductive technology (27, 28). Similarly, increased DNA fragmentation was also observed in decreased semen quality in men with COVID-19, together with an imbalance of oxidation and anti-oxidation (29).

For cell cycle-associated signaling, it is very reasonable to be enriched here. SARS-CoV-2-facilitated host cell death is inseparable from nucleocapsid (N) protein-dependent G1 cell cycle arrest mechanism, and this N protein is also involved in viral replication and transcription, and many stages of the viral life cycle (30). Interestingly, much research on SARS-CoV-2 infection and other comorbidities, such as myocarditis (31), cardiac injury (32), and Alzheimer’s disease (33), also found that genetic alteration of cell cycle-related genes was very significant in the pathogenesis. Ubiquitination signaling is crucial for immunity to limit the spread of pathogens, thus, it plays a critical role in the viral pathogenicity of SARS-CoV-2. For example, aberrant USP29 deubiquitylation could enhance the virulence of SARS-CoV-2 by preventing proteasome degradation of ORF9b (34). One of the underlying mechanisms why SARS-CoV-2 infection can promote autoinflammatory disease was that SARS-CoV-2 affects UBA1 and ubiquitination in an inappropriate way (35). Nevertheless, in addition to DNA damage and repair, no findings of cell cycle-associated, and ubiquitination-associated signaling were reported in COVID-19-associated MI, which deserves further exploration.

One of the highlights of the study was to identify and validate hub CORGs through multi-omic analysis. The hub CORGs should meet the requirements of high connectivity score (MCC, EPC, BottleNeck, EcCentricity, Closeness, and Radiality) in the PPI network first. Then, the hub CORGs must show good diagnostic performance not only in the initial COVID-19 and MI datasets, but also in two independent validation sets. Third, the significance of hub CORGs was evaluated in the expression levels of sperm samples from COVID-19 patients with MI, and healthy controls. The first hub CORG, CIB1 (Calcium And Integrin-Binding Protein 1), encodes a member of the calcium-binding superfamily with an EF-hand domain. It has been revealed to have a wide variety of roles in cellular processes, including cell differentiation, proliferation, migration, adhesion, and survival (36). CIB1 has the implication in micropinocytosis-mediated virus entry in dermal endothelial cells (37), which may imply its role in virus infiltration of SARS-CoV-2. In terms of fertility, Yuan et al. manifested that CIB1 is required for cell cycle of spermatogenic cells, and/or differentiation of Sertoli cells (38). Our results showed the significant change of CIB1 in sequencing and experimental data, and that it may have the potential as a biomarker for COVID-19-induced spermatogenesis impairment.

EIF3B (Eukaryotic Translation Initiation Factor 3 Subunit B) acts as the second hub CORG with a significant difference in the qRT-PCR result. It is an important RNA-binding component of the eukaryotic translation initiation factor 3 (EIF-3) complex. Current evidence found that EIF3B propels the internal ribosome entry site (IRES) element-mediated viral translation initiation and viral translational termination/reinitiation (39, 40). Furthermore, EIF3B was demonstrated as a critical regulator of cell cycle in cancer progression (41–43). Therefore, there is a possibility that EIF3B affects the viral translation of SARS-CoV-2 and the cell fate of host cell. The third hub CORG is ENTPD6 (Ectonucleoside Triphosphate Diphosphohydrolase 6), which contains four apyrase-conserved regions which are characteristic of E-type nucleotidases. A genome-wide association study on non-obstructive azoospermia (NOA) indicated that ENTPD6 may be a novel meiosis-associated gene responsible for NOA after functional screening, which suggested the role of this gene in spermatogenesis. However, the difference in the expression of ENTPD6 in **Figure 9** was not statistically significant, thus, the value of ENTPD6 in COVID-19-associated MI deserves further verification.

Among one hundred and thirty-five pharmacologic agent candidates targeting CORGs, we found four candidates (ascorbic acid, biotin, caffeine, and L-cysteine) with supporting evidence in *in vitro* or *in vivo* studies. A high dose of ascorbic acid was shown to be favorable to mitigating symptoms of respiratory tract infection and mortality for COVID-19 patients (44). And its antioxidant properties make it a good therapeutic agent for impaired sperm quality (45). Several investigations confirmed the benefit of supplementation of caffeine or L-cysteine in clinical susceptibility, presentation, and prognosis for COVID-19 patients, possibly through blocking the virus from penetrating host cells or decreasing viral replication by preventing the action of 3-chymotrypsin-like proteases (46, 47). What’s more, adding caffeine had a beneficial effect on sperm motility and metabolism to some extent (48) Biotin was regarded as a protector during cryopreserving human spermatozoa, improving the motility and lifetime of frozen-thawed spermatozoa (49). It is a pity that the relationship between biotin and COVID-19 remains elusive. Based on the findings, these four pharmacologic agent candidates have the potential for improving COVID-19-associated MI, and a clinical trial is needed to verify their therapeutic effect.

It is noted that immunomodulation permeates the entire process of virus invasion, replication, and clearance. According to the immunological results displayed in **Figure 7**, obvious differences in the levels of multiple immune cells were observed between the COVID-19 and control samples. In the next step, we found three types of immune cells (CD8+ T cells, monocytes, and macrophages M0) had a close relationship with hub CORGs. During the acute and convalescent stages of COVID-19, SARS-CoV-2-specific CD8+ T cell responses were activated, leading to protective immune responses against SARS-CoV-2 (50). Monocytes are phagocytic innate immune cells that circulate in the circulation, and macrophages exist in almost all tissues of the body, which ingest infectious cells, toxic agents, and other microscopic particles (51). Mylvaganam et al. (52) showed that high levels of monocyte/macrophage and cytokines in acute COVID-19, persistently resulting in the activation of fibroblastic signaling and further leading to pulmonary fibrosis. In addition, Zhong et al. (53) indicated that CD8+ T cell was dominantly enriched in immune infiltration of patients with non-obstructive azoospermia. Cytokines generated from monocytes and macrophages were detected in human semen. Taken together with our data, the levels of CD8+ T cell and monocyte were positively consistent with the expression of hub CORGs in COVID-19 samples, while the level of macrophage M0 (non-activated state) had a negative correlation. It is reasonable to speculate that hub CORGs regulate the activity of the aforementioned immune cells and affects the reproductive function after SARS-CoV-2 infection.

It is the first time for us to mine the genetic interrelationship between COVID-19 and MI. There are some limitations in the study. First, diverse sequencing platforms and different ethnic subjects in the genetic data have influence on the accuracy of our analysis to some extent. Then, although multi-omics analysis, including external validation of independent data and experimental measurement, was carried out to identify and validate the importance of CORGs, biases were still present across analyses due to the limited sample size. A large cohort study is needed to confirm our findings. Third, it is of great importance to use the same tissue source for bioinformatics studies to reduce heterogeneity. For example, using semen samples from COVID-19 patients with MI to perform bioinformatic analyses would obtain more reliable results. Last but not least, in-depth pathogenic mechanisms of hub CORGs, promising pathway and protein alteration were not well explored at this time, and it is significant to be clarified in our subsequent investigations.

## 5 Conclusion

In summary, we comprehensively explored the molecular profiles and biological mechanisms in male infertility during COVID-19 pandemic. A hub gene signature containing ENTPD6, CIB1, and EIF3B was uncovered and validated, together with its relevance to immune response, transcriptional regulation, and expression condition in semen. Our study provided new clues for the pathogenesis of these two diseases and shared emerging potential biomarkers for COVID-19-associated MI.

## Data availability statement

The raw data supporting the conclusions of this article will be made available by the authors, without undue reservation.

## Ethics statement

The studies involving human participants were reviewed and approved by Huazhong University of Science and Technology’s institutional research ethics committee. The patients/participants provided their written informed consent to participate in this study.

## Author contributions

YC, PY and CL contributed to the research design, data processing and statistical analysis. KL contributed to participant recruitment and collection of medical record. TS contributed to semen processing and experimental measurement. All authors contributed to the article and approved the submitted version.

## Supplementary material

Supplementary Figure 1 Heatmap of DEGs in the two independent validation sets. **(A)** Heatmap depicts the DEGs between the COVID-19 and control groups in an independent validation set (GSE147507). **(B**) Heatmap depicts the DEGs between the MI and control groups in an independent validation set (GSE26881). DEGs, differentially expressed genes; GSE, GEO Series; MI, male infertility.

Supplementary Figure 2 Expression patterns of two hub CORGs (CIB1 and EIF3B) in the normal testicular tissues. **(A)** Immunohistochemistry result of CIB1 in the HPA database (scale bars = 50μm and 200 μm). **(B)** Immunohistochemistry result of EIF3B (scale bars = 50μm and 200 μm).
