# Supplementary figures and images for: Exploration of the common genetic landscape of COVID-19 and male infertility
Source: Front Immunol. 2023 Mar 20;14:1123913. doi: 10.3389/fimmu.2023.1123913 (PMC10067640; doi:10.3389/fimmu.2023.1123913)

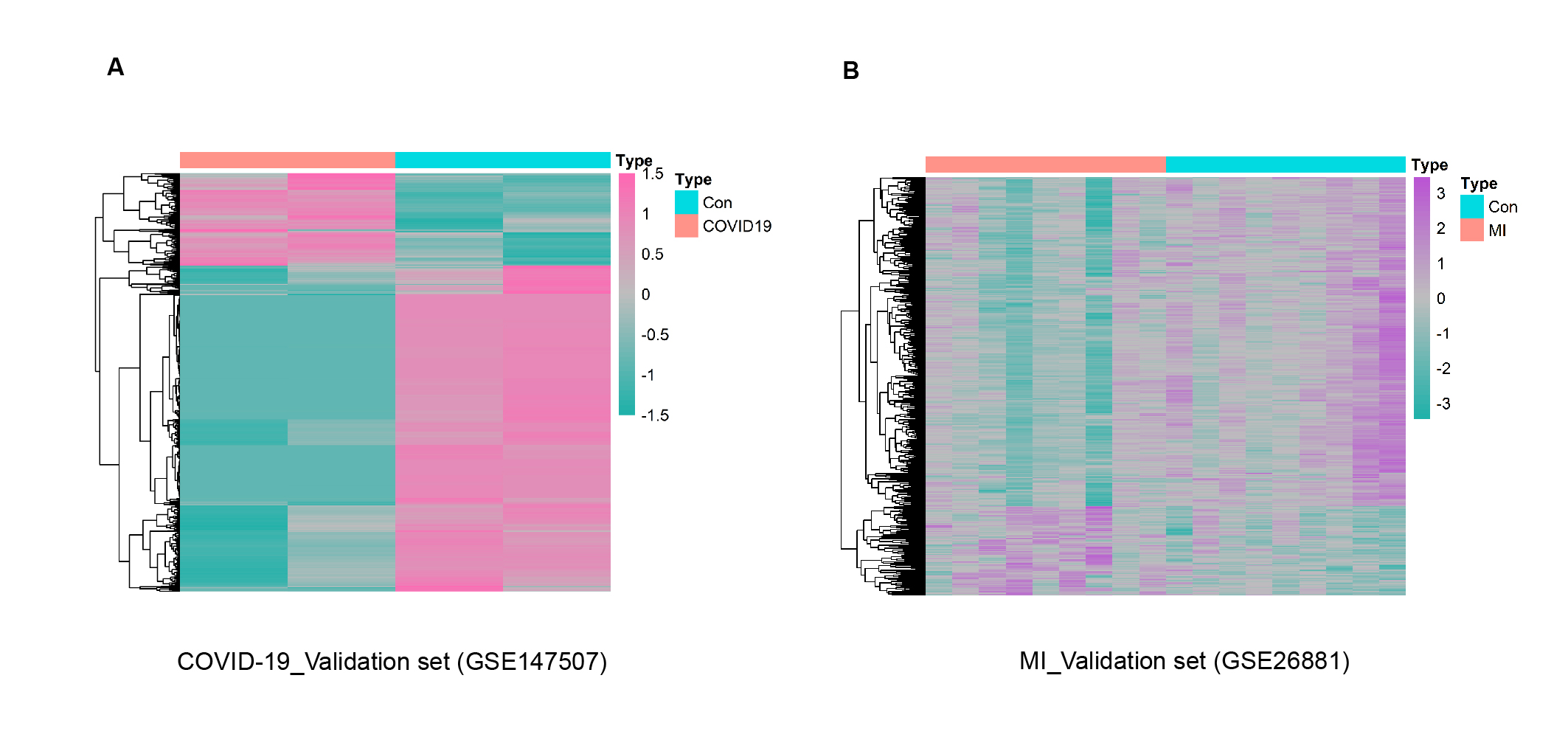

Supplement: Supplementary Figure 1 — Heatmap of DEGs in the two independent validation sets. (A) Heatmap depicts the DEGs between the COVID-19 and control groups in an independent validation set (GSE147507). (B) Heatmap depicts the DEGs between the MI and control groups in an independent validation set (GSE26881). DEGs, differentially expressed genes; GSE, GEO Series; MI, male infertility. [file Image_1.jpeg]

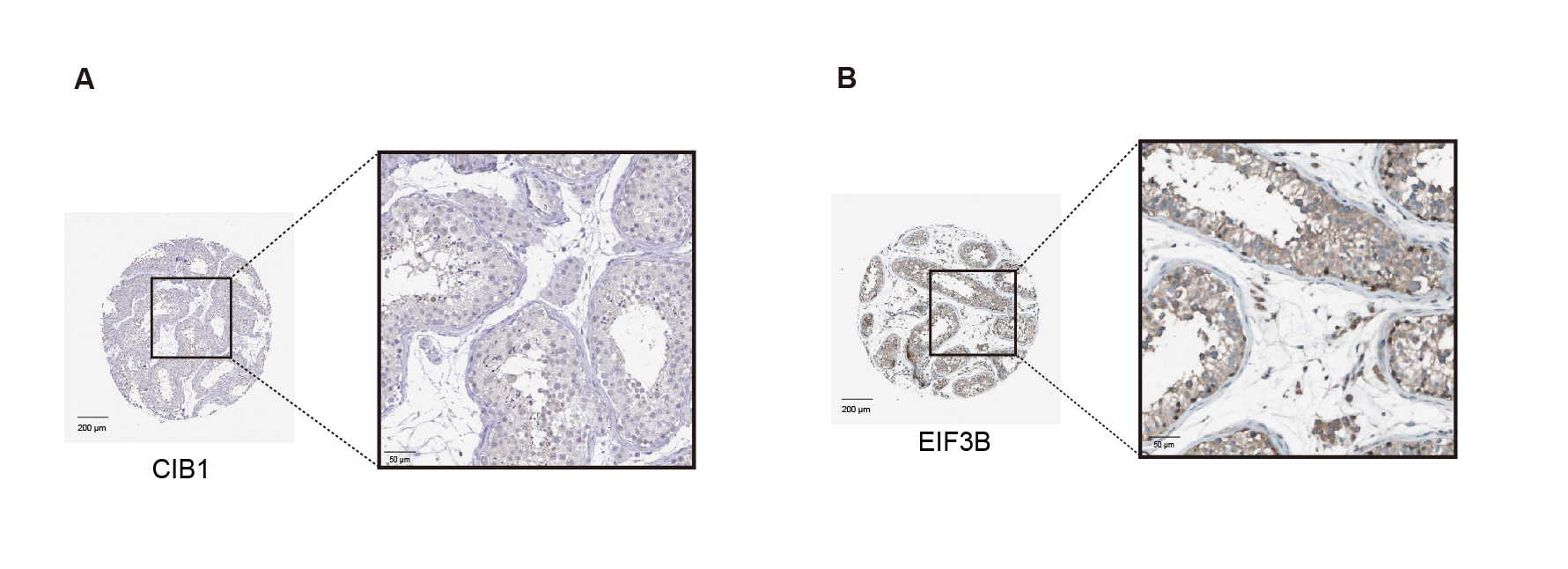

Supplement: Supplementary Figure 2 — Expression patterns of two hub CORGs (CIB1 and EIF3B) in the normal testicular tissues. (A) Immunohistochemistry result of CIB1 in the HPA database (scale bars = 50μm and 200 μm). (B) Immunohistochemistry result of EIF3B (scale bars = 50μm and 200 μm). [file Image_2.jpeg]
