# Supplementary material for: Exploration of the common genetic landscape of COVID-19 and male infertility
Source: Front Immunol. 2023 Mar 20;14:1123913. doi: 10.3389/fimmu.2023.1123913 (PMC10067640; doi:10.3389/fimmu.2023.1123913)
Supplement: Supplementary file 3 [file Table_1.docx]

**Supplementary table 1**

Primer sequence in the qRT-PCR analysis.

| Gene | Primer type | Sequence/Target sequence |
| --- | --- | --- |
| ENTPD6 (human) | Forward | 5’- GGACATTCCGTTCGACTTCTG -3’ |
| ENTPD6 (human) | Reverse | 5’- CTACAAGGAAAGGCGATGCTT -3’ |
| CIB1 (human) | Forward | 5’- CACGGCTTAGTGCGTCTGAG -3’ |
| CIB1 (human) | Reverse | 5’- AAAGTCTGGAGAACGGGAGAT -3’ |
| EIF3B (human) | Forward | 5’- GGACCCGACCGACTTGAGA -3’ |
| EIF3B (human) | Forward | 5’- TTGACCCGGAATGTGTGCTG -3’ |
| GAPDH (human) | Forward | 5’-TGGTCACCAGGGCTGCTTTTA-3’ |
| GAPDH (human) | Reverse | 5’-CATCGCCCCACTTGATTTTG-3’ |

qRT-PCR: quantitative real-time polymerase chain reaction; ENTPD6: Ectonucleoside Triphosphate Diphosphohydrolase 6; CIB1: Calcium And Integrin Binding 1; EIF3B: Eukaryotic Translation Initiation Factor 3 Subunit B; GAPDH: glyceraldehyde-3-phosphate dehydrogenase.
