# Supplementary material for: Exploration of the common genetic landscape of COVID-19 and male infertility
Source: Front Immunol. 2023 Mar 20;14:1123913. doi: 10.3389/fimmu.2023.1123913 (PMC10067640; doi:10.3389/fimmu.2023.1123913)
Supplement: Supplementary file 4 [file Table_2.docx]

**Supplementary table 2**

CORGs with AUC > 0.9 in the study.

| Gene | AUC in COVID-19 dataset | Gene | AUC in MI dataset |
| --- | --- | --- | --- |
| RNF7 | 0.96 | RNF7 | 0.90 |
| RPA1 | 1.00 | UBE2G1 | 0.92 |
| UBXN7 | 0.94 | ATG7 | 0.93 |
| POLD2 | 0.94 | EPS15 | 0.93 |
| COMMD3 | 1.00 | PSMB1 | 0.90 |
| RAD51 | 0.92 | AKT3 | 0.92 |
| UBE2G1 | 1.00 | STIL | 0.90 |
| UBE2E2 | 1.00 | PDE3B | 0.92 |
| CHD1L | 0.98 | TBCD | 0.94 |
| RBBP8 | 1.00 | YWHAE | 0.93 |
| POLR2J | 1.00 | ENTPD6 | 0.94 |
| DCAF10 | 0.94 | EIF3B | 0.90 |
| CTDP1 | 1.00 | PPP6R3 | 0.92 |
| UBR1 | 1.00 | ZEB1 | 0.92 |
| PRKDC | 0.96 | ATF6 | 0.90 |
| ATG7 | 1.00 | RBP4 | 0.90 |
| WDR48 | 0.96 | C2orf69 | 0.90 |
| CDK12 | 1.00 | CIB1 | 0.94 |
| RPRD1B | 0.98 |  |  |
| PIK3C3 | 1.00 |  |  |
| ITCH | 0.92 |  |  |
| AQR | 1.00 |  |  |
| GSK3B | 1.00 |  |  |
| KIF14 | 1.00 |  |  |
| BOP1 | 1.00 |  |  |
| BTAF1 | 1.00 |  |  |
| DDX10 | 0.92 |  |  |
| RNF11 | 1.00 |  |  |
| CYLD | 0.98 |  |  |
| PLCB2 | 1.00 |  |  |
| PPP2R5E | 0.98 |  |  |
| RANBP2 | 0.92 |  |  |
| AKT3 | 1.00 |  |  |
| FGF2 | 0.98 |  |  |
| STIL | 0.92 |  |  |
| KIF2A | 1.00 |  |  |
| RAP1GDS1 | 1.00 |  |  |
| ITGB3BP | 0.96 |  |  |
| SYNRG | 1.00 |  |  |
| PDE3B | 1.00 |  |  |
| RPL3L | 1.00 |  |  |
| BRAF | 0.94 |  |  |
| ACTR2 | 1.00 |  |  |
| NUP160 | 1.00 |  |  |
| ENTPD6 | 0.98 |  |  |
| EIF3B | 1.00 |  |  |
| TBC1D7 | 1.00 |  |  |
| YTHDC2 | 1.00 |  |  |
| CSPP1 | 0.92 |  |  |
| SGSM2 | 1.00 |  |  |
| MOV10L1 | 1.00 |  |  |
| CENPJ | 0.96 |  |  |
| TDRD3 | 1.00 |  |  |
| TAF2 | 1.00 |  |  |
| EIF4E1B | 1.00 |  |  |
| RFWD3 | 0.92 |  |  |
| SEC24B | 0.94 |  |  |
| RRAGC | 0.98 |  |  |
| CRKL | 1.00 |  |  |
| TMPO | 1.00 |  |  |
| ZEB1 | 0.98 |  |  |
| RARS | 0.98 |  |  |
| LRRC28 | 1.00 |  |  |
| MAN1A2 | 0.94 |  |  |
| STAU1 | 0.94 |  |  |
| STAG1 | 1.00 |  |  |
| INTS2 | 1.00 |  |  |
| CRY1 | 1.00 |  |  |
| WDPCP | 1.00 |  |  |
| DAP3 | 0.92 |  |  |
| METAP2 | 1.00 |  |  |
| RC3H2 | 1.00 |  |  |
| TBC1D12 | 0.96 |  |  |
| PIGN | 1.00 |  |  |
| MTCH2 | 0.96 |  |  |
| MAPK6 | 1.00 |  |  |
| STRN3 | 1.00 |  |  |
| FNTA | 0.96 |  |  |
| SLC2A1 | 1.00 |  |  |
| TNFSF11 | 0.96 |  |  |
| RBL1 | 0.94 |  |  |
| WDR27 | 1.00 |  |  |
| ZRANB3 | 1.00 |  |  |
| METAP1 | 0.98 |  |  |
| SHOC2 | 1.00 |  |  |
| ISCU | 1.00 |  |  |
| RBP4 | 1.00 |  |  |
| ATP6V1D | 1.00 |  |  |
| NRIP1 | 1.00 |  |  |
| AIM2 | 0.96 |  |  |
| CD1D | 0.96 |  |  |
| C2orf69 | 1.00 |  |  |
| ZNF410 | 1.00 |  |  |
| TRIM47 | 1.00 |  |  |
| PNPLA8 | 1.00 |  |  |
| CTF1 | 0.94 |  |  |
| GALC | 0.92 |  |  |
| ZNF292 | 1.00 |  |  |
| TCAP | 1.00 |  |  |
| CIB1 | 1.00 |  |  |

CORGs: COVID-19-related differentially expressed genes; MI: male infertility; AUC: area under the curve.
