# Supplementary material for: Exploration of the common genetic landscape of COVID-19 and male infertility
Source: Front Immunol. 2023 Mar 20;14:1123913. doi: 10.3389/fimmu.2023.1123913 (PMC10067640; doi:10.3389/fimmu.2023.1123913)
Supplement: Supplementary file 5 [file Table_3.docx]

**Supplementary table 3**

Demographic characteristics and laboratory tests of patients with COVID-19 and MI.

|  | COVID-19 and MI | Control | P value |
| --- | --- | --- | --- |
| Age (year) | 28.71±5.19 | 29.86±2.80 | 0.617 |
| BMI (kg m^-2^) | 21.03±1.67 | 22.64±2.54 | 0.187 |
| Semen volume (ml) | 3.19±0.9 | 3.11±1.77 | 0.926 |
| Semen pH | 7.37±0.2 | 7.24±0.11 | 0.161 |
| Sperm total number (10^6^) | 113.2±101.9 | 235.7±143.0 | 0.090 |
| Sperm concentration (10^6^ ml^-1^) | 33.81±25.9 | 83.49±53.47 | 0.047 |
| Sperm motility (%) | 27.26±13.99 | 59.07±4.06 | < 0.0001 |
| Sperm progressive motility (%) | 27.54±12.74 | 48.06±5.11 | < 0.01 |
| Normal sperm morphology (%) | 9.73±4.71 | 12.62±2.18 | 0.167 |
| Serum FSH (mIU ml^-1^) | 4.57±1.6 | - | - |
| Serum LH (mIU ml^-1^) | 3.02±1.29 | - | - |
| Serum T (ng ml^-1^) | 3.03±0.72 | - | - |
| Serum E_2_ (pg ml^-1^) | 48.14±13.62 | - | - |
| History of varicocele | Neg | Neg | - |
| History of inflammation and infection of the genitourinary tract | Neg | Neg | - |
| History of scrotal trauma | Neg | Neg | - |
| Exposure to radiation, toxic and harmful substances, and drugs | Neg | Neg | - |
| Smoking | Neg | Neg | - |
| Alcohol abuse | Neg | Neg | - |

MI: male infertility; BMI: body mass index; FSH: follicle‑stimulating hormone (normal range: 1.27-19.26 mIU ml^-1^); LH: luteinizing hormone (normal range: 1.24-8.62 mIU ml^-1^); T: testosterone (normal range: 1.75-7.81 ng ml^-1^); E_2_: estradiol (normal range: ≤ 38.95 pg ml^-1^); Neg: negative.
